# Phenotyping of Fontan‐Associated Renal Physiology and Disease

**DOI:** 10.1002/cph4.70116

**Published:** 2026-03-10

**Authors:** Gaston van Hassel, Jozine M. ter Maaten, Iris E. Beldhuis, Eryn T. Liem, Bastiaan Zwart, Stephan J. L. Bakker, Rolf M. F. Berger, Joost P. van Melle

**Affiliations:** ^1^ University of Groningen, Center for Congenital Heart Diseases, Department of Paediatric Cardiology Beatrix Children's Hospital, University Medical Center Groningen Groningen the Netherlands; ^2^ Department of Cardiology University of Groningen, University Medical Center Groningen Groningen the Netherlands; ^3^ Center for Congenital Heart Diseases, Department of Cardiology University of Groningen, University Medical Center Groningen Groningen the Netherlands; ^4^ University of Groningen, Department of Internal Medicine, Division of Nephrology University Medical Center Groningen Groningen the Netherlands

## Abstract

**Background:**

Kidney dysfunction is prevalent but insufficiently understood in Fontan patients. We aimed to investigate Fontan‐Associated Renal Disease (FARD) through comprehensive phenotyping using biomarkers of renal function and injury, as well as ultrasound assessment of intrarenal hemodynamics.

**Methods:**

We prospectively collected data on serum creatinine and cystatin C levels, and 24‐h urine creatinine clearance and albumin excretion in 25 Fontan patients, comparing them to healthy age‐ and sex‐matched controls. Additionally, we measured intrarenal venous and arterial flow patterns using Doppler ultrasonography.

**Results:**

Median age was 28 (Q1–Q3: 19–35) years, with 48% female. Compared to controls, Fontan patients had similar creatinine‐based eGFR (eGFR_cr_) (114 vs. 106 mL/min/1.73 m^2^; *p* = 0.2), lower cystatin C‐based eGFR (eGFR_cys_) (96 vs. 106 mL/min/1.73 m^2^; *p* = 0.045), and lower 24‐h urinary creatinine excretion (10.0 vs. 13.7 mmol/24 h; *p* < 0.001). Additionally, patients had higher 24‐h urine albumin excretion (14 vs. 2 mg/24‐h; *p* < 0.001). In patients, both eGFR_cys_ and urine albumin correlated with age (*r* = −0.56 and *r* = 0.44, respectively; *p* < 0.05). Twenty‐‐four out of 25 patients and all controls had continuous intrarenal venous flow patterns. Venous impedance and arterial renal resistance indices were higher in patients than controls (0.44 vs. 0.14 and 0.67 vs. 0.60, respectively; *p* < 0.05).

**Conclusion:**

FARD involves a progressive decline in kidney function and glomerular injury. eGFR_cr_ masks kidney dysfunction in Fontan patients due to reduced muscle mass. Fontan intrarenal hemodynamics demonstrate decreased venous and arterial compliance. Moreover, continuous intrarenal venous flow persists in Fontan patients.

## Graphical abstract

In patients with a Fontan circulation, both kidney function and glomerular injury progressively decline with age, with a stronger decline than in healthy individuals. Additionally, intrarenal haemodynamics in patients with a Fontan circulation demonstrate decreased venous and arterial compliance, evident through elevated venous impedance and renal resistance indices.

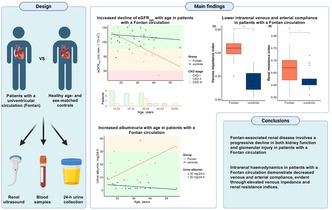

## 1 Introduction

The Fontan circulation is the surgical treatment for children born with a functionally univentricular heart in whom biventricular repair is not suitable (Fontan and Baudet 1971). By directly connecting the caval veins to the pulmonary artery, the Fontan circulation bypasses the sub‐pulmonary ventricle. Consequently, systemic venous return relies on postcapillary inertia, while the absence of a sub‐pulmonary ventricular pump to overcome pulmonary vascular resistance induces a state of chronically elevated central venous pressure (CVP) and reduced cardiac output (CO) (Gewillig and Brown 2016). Over time, functional decline of this circulation is inevitable and will lead to a state of multiorgan complications, including renal disease (Wolff et al. 2017).

Fontan‐associated renal disease (FARD) is prevalent amongst 10%–50% of patients with a Fontan circulation and progressively worsens over time, with the rate of deterioration linked to an increased risk of all‐cause mortality (Hassel et al. 2024; Zafar et al. 2020; Alsaied et al. 2017). Due to its association with prognosis and impact on quality of life, accurate assessment of kidney function is crucial in patients with a Fontan circulation (Hassel et al. 2024; Zafar et al. 2020). However, data on renal diagnostic parameters specific to Fontan physiology remain scarce. It remains unclear at what point renal adaptations become detectable and which diagnostic markers are most appropriate. Therefore, comprehensive phenotyping and comparison with healthy age‐ and sex‐matched controls are essential.

This study aims to characterize FARD by comprehensive renal phenotyping, including the assessment of serum biomarkers and 24‐h urine biomarkers for glomerular and tubular damage, as well as Doppler ultrasound of the kidney to analyze intrarenal venous and arterial flow patterns. The outcomes will be compared to those of healthy, age‐ and sex‐matched controls to identify Fontan‐specific characteristics and to unravel potential pathophysiological mechanisms.

## 2 Methods

### 2.1 Study Population, Design, and Data Collection

In this case–control study, we aimed to phenotype Fontan‐associated renal physiology and disease through serum and 24‐h urine biomarkers, as well as intrarenal flow patterns as assessed with ultrasound Doppler imaging, in both patients with a Fontan circulation and in healthy age‐ and sex‐matched controls. Patients were prospectively enrolled in a tertiary center for congenital heart disease (University Medical Centre Groningen, The Netherlands) between June 2024 and January 2025. In general, patients were eligible for enrolment if they were older than 12 years of age, able to provide written informed consent (or their parents/guardians), were not receiving intermittent or continuous hemodialysis and were not pregnant. Healthy controls were recruited through recruitment brochures posted in the hospital and the University of Groningen, or as siblings of patients with a Fontan circulation. The same inclusion criteria as for the patients were applied for the healthy volunteers; however, for the control group we excluded individuals with known cardiac or renal disease and those using nephrotoxic medication. Patients and healthy controls were age‐ and sex‐matched, with the age difference between matched pairs not exceeding 2 years. A predefined total number of 25 patients with a Fontan circulation and 25 controls were enrolled. Demographic, clinical, and cardiac‐related characteristics were extracted from patient records (patients) or asked prior to the study visit (controls). The study was conducted in accordance with the Declaration of Helsinki. The study protocol was approved by the institutional review board (METc 2022530), and all participants provided written informed consent.

### 2.2 Laboratory Measurements and Urine Sampling

Venous blood samples were drawn during the study visit. Hematological measurements were obtained using standard laboratory techniques. Estimated glomerular filtration rate (eGFR) was calculated using the Chronic Kidney Disease Epidemiology Collaboration equation (CKD‐EPI) for individuals aged 18 or older and the bedside Schwartz and CKiD equation for individuals younger than 18 years old using serum creatinine (eGFR_cr_) and cystatin C (eGFR_cys_). Urine albumin—reflecting impairment of the barrier function of the glomerulus—was measured from the collected 24‐h urine samples (Levey et al. 2022). Also, cystatin C, typically fully reabsorbed in the proximal tubules, and liver‐type fatty acid binding protein (L‐FABP), released from the tubules in response to injury, were measured in 24‐h urine as markers of chronic tubular damage (Yang et al. 2025; Mitsides et al. 2023; Conti et al. 2006).

### 2.3 Renal Ultrasound Assessment

Intrarenal Doppler flow patterns, both arterial and venous, were obtained using a commercially available system with a convex transducer frequency range of 2.5–5.0 MHz (GE). Resting images were taken in the left semilateral decubitus position where the right kidney was recorded. In case of unsatisfactory image quality, the left kidney was recorded in the opposite position. An electrocardiogram (ECG) signal was simultaneously recorded by the ultrasound system. All measurements were averaged over 3 cardiac cycles during sinus rhythm.

**FIGURE 1:**
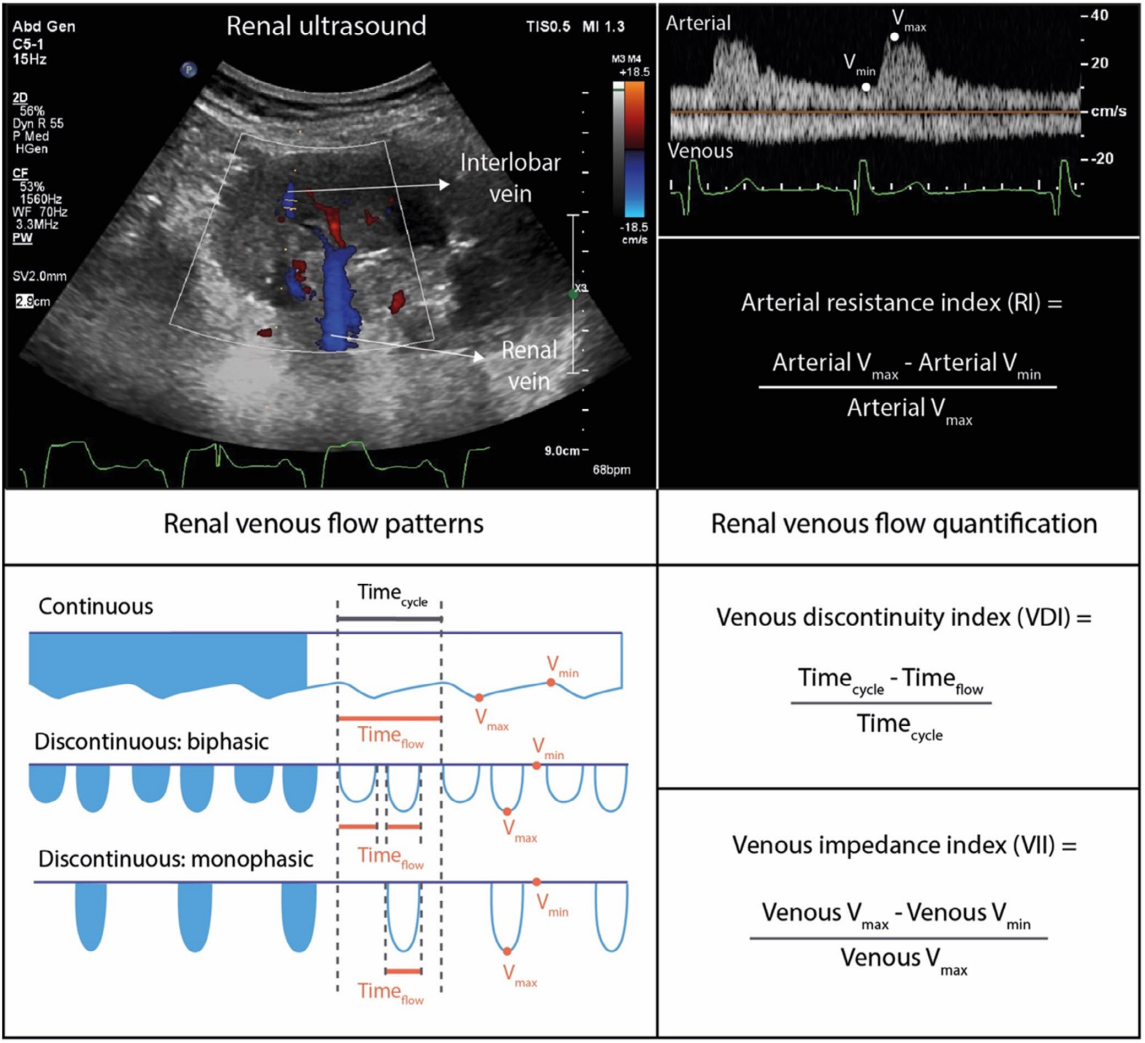
Assessment and calculation of renal ultrasound parameters. RRI = intrarenal resistance index, VDI = venous discontinuity index, VII = venous impedance index. Reprinted from: The Effect of Decongestion on Intrarenal Venous Flow Patterns in Patients With Acute Heart Failure, Journal of Cardiac Failure, Volume 27, Issue 1, January 2021 (with permission).

Intralobular renal venous and arterial flow patterns were analyzed to assess perfusion and vascular compliance. Typical examples of intrarenal Doppler flow patterns as previously described for non‐Fontan patients are depicted in Figure 1 (Maaten et al. 2021). As shown, in normal conditions, intrarenal venous flow is continuous with a small varying amplitude during the cardiac cycle. The venous impedance index (VII) can be calculated as the difference between peak maximum flow velocity and nadir flow velocity, divided by the peak velocity. As CVP increases, the amplitude variation in intrarenal venous flow grows until the flow becomes discontinuous (Maaten et al. 2021). For subjects with discontinuous flow, the venous discontinuity index can be calculated as the percentage of time without flow during a cardiac cycle (Maaten et al. 2021). Additionally, intrarenal arterial flow and compliance can be assessed through the renal resistance index (RRI). The RRI is determined by subtracting diastolic flow velocity from maximum flow velocity and dividing by maximum velocity.

### 2.4 Statistical Analysis

Data are presented as mean ± standard deviation or median (Q1–Q3), depending on distribution and as number (percentage) for categorical variables. Normality was evaluated by visual inspection of histograms and Q‐Q plots. Differences between patients with a Fontan circulation and controls were analyzed using a Mann–Whitney *U* test for both normally distributed and skewed variables. The *χ* ^2^ test was used for categorical variables.

Univariable cross‐sectional analyses for the associations between kidney function, markers of glomerular and tubular damage, intrarenal hemodynamics and patient characteristics were performed using Spearman's ρ. Adjusted multivariable linear regression analyses were performed with eGFR_cys_ and 24‐h urine albumin as dependent variables and age, sex, RRI, and VVI as independent variables. Normality of the residuals was evaluated by visual inspection of Q‐Q plots, where variables were binary logarithmically transformed (log_2_) if necessary to reach assumptions for linear regression.

All analyses were performed using the R statistical software (version 4.3.2). Statistical significance was set at a 2‐tailed probability level of < 0.05.

## 3 Results

### 3.1 Baseline Characteristics

Twenty‐five patients with a Fontan circulation and 25 healthy subjects were included in the study (Table 1). The median age of the entire population was 28 (19–35) years. Four (16%) patients were younger than 18 years old, and 48% of the study population was female (*n* = 24). Patients with a Fontan circulation were on average smaller than the healthy controls (172 ± 14 vs. 179 ± 10 cm, *p* = 0.049). The majority of the Fontan patients were palliated with an extracardiac conduit (*n* = 13), followed by a lateral tunnel connection (*n* = 8), atrial connection (*n* = 3), and a bidirectional cavopulmonary connection with a bidirectional Glenn anastomosis (*n* = 1). Median time since Fontan completion was 21 (12–31 years). Patients had on average higher hemoglobin (9.6 vs. 9.0 mmol/L, *p* = 0.02), NT‐proBNP (210 vs. 28 ng/L, *p* < 0.001), aspartate transferase (AST) (31 vs. 22 U/L, *p* < 0.001), alanine transaminase (ALT) (28 vs. 16 U/L, *p* < 0.001), and gamma‐glutamyltransferase (γGT) (78 vs. 17 U/L, p < 0.001) levels than controls. Lastly, patients had lower creatinine kinase (CK) levels (82 vs. 112 U/L, *p* = 0.02).

**TABLE 1:** Clinical characteristics.

	Patients (*n* = 25)	Controls (*n* = 25)	*p*
Age, years	27 (19–35)	28 (19–35)	0.9
Height, cm	172 ± 14	179 ± 10	0.049
Weight, kg	71 ± 15	70 ± 12	0.7
BSA, m^2^	1.67 (1.89–2.07)	1.74 (1.86–1.97)	0.7
Type of Fontan (*n*(%))			
Atrial connection	3 (12%)	—	
Lateral tunnel	8 (32%)	—	
Extracardiac conduit	13 (52%)	—	
Bidirectional CPC	1 (4%)	—	
Blood			
Hb, mmol/L	9.64 ± 0.91	9.04 ± 0.80	0.02
Ht, L/L	0.46 ± 0.04	0.43 ± 0.03	0.002
MCV, fl	88.9 ± 4.9	88.8 ± 3.2	0.9
Sodium, mmol/L	140 (139–141)	140.00 (139–141)	0.8
Potassium, mmol/L	4.0 (3.8–4.3)	4.1 (3.9–4.2)	0.8
NT‐proBNP, ng/L	210 (62–376)	28 (20–38)	< 0.001
CK, μ/L	82 (60–101)	112 (79–142)	0.02
AST, μ/L	31.0 (25.0–33.0)	22.0 (20.0–27.0)	< 0.001
ALT, μ/L	28 (21–32)	16 (12–21)	< 0.001
γGT, μ/L	78 (54–140)	17 (14–20)	< 0.001
Medication (*n* (%))			
ACEi/ARB	4 (16%)	0 (0%)	0.1
Beta‐blockers	12 (48%)	0 (0%)	< 0.001
MRA	6 (24%)	0 (0%)	0.03
Loop diuretics	3 (12%)	0 (0%)	0.2
Sildenafil	5 (20%)	0 (0%)	0.06

Abbreviations: γGT = γ‐glutamyltransferase, ACEi = angiotensin converting enzyme inhibitor, ALT = alanine transaminase, ARB = angiotensin II receptor blocker, AST = aspartate transaminase, BSA = body surface area, CK = creatinine‐kinase, CPC = cavopulmonary connection, Hb = hemoglobin, Ht = hematocrit, MCV = mean corpuscular volume, NT‐proBNP = *N*‐terminal prohormone of brain natriuretic peptide.

### 3.2 Kidney (dys)Function

There was no significant difference in serum creatinine levels (70 vs. 77 μmol/L; *p* = 0.3) and eGFR_cr_ (114 vs. 106 mL/min/1.73 m^2^; *p* = 0.2) between patients and controls, but patients had lower 24‐h urinary creatinine excretion (10.0 vs. 13.7; *p* < 0.001) (Table 2). Patients had significantly higher cystatin C levels (0.89 vs. 0.85 mg/L; *p* = 0.046) and lower eGFR_cys_ (96 vs. 106 mL/min/1.73m^2^; *p* = 0.045) than controls.

**TABLE 2:** Serum and 24‐h biomarkers of kidney function and injury.

	Patients (*n* = 25)	Controls (*n* = 25)	*p*
Serum biomarkers			
Creatinine, μmol/L	70 ± 20	77 ± 21	0.3
eGFR_cr_, mL/min/1.73 m^2^	114 ± 24	106 ± 21	0.2
Cystatin C, mg/L	0.89 (0.83–0.99)	0.85 (0.74–0.89)	0.046
eGFR_cys_, mL/min/1.73 m^2^	96 ± 21	106 ± 13	0.045
BUN, mg/dL	15.4 (12.6–17.6)	14.7 (11.0–15.9)	0.1
24‐h urine biomarkers			
Creatinine, mmol/24 h	10.0 (8.5–12.7)	13.7 (10.2–17.6)	< 0.001
Creatinine clearance, mL/min	94 (81–101)	120 (100–149)	0.036
Albumin, mg/24 h	14 (5–29)	2 (2, 4)	< 0.001
Sodium, mmol/24 h	120 (88–154)	144 (112–199)	0.1
Cystatin C, mg/24 h	0.034 (0.019–0.049)	0.037 (0.027–0.043)	0.5
L‐FABP, pg/24 h	2402 (2134–2670)	2401 (2021–3632)	0.6

*Note:* Albuminuria was defined as urine albumin > 30 mg/24 h.

Abbreviations: BUN = blood urea nitrogen, eGFR_cr_ = creatinine‐based estimated glomerular filtration rate, eGFR_cys_ = cystatin C based estimated glomerular filtration rate, L‐FABP = liver‐type fatty acid‐binding protein.

**FIGURE 2:**
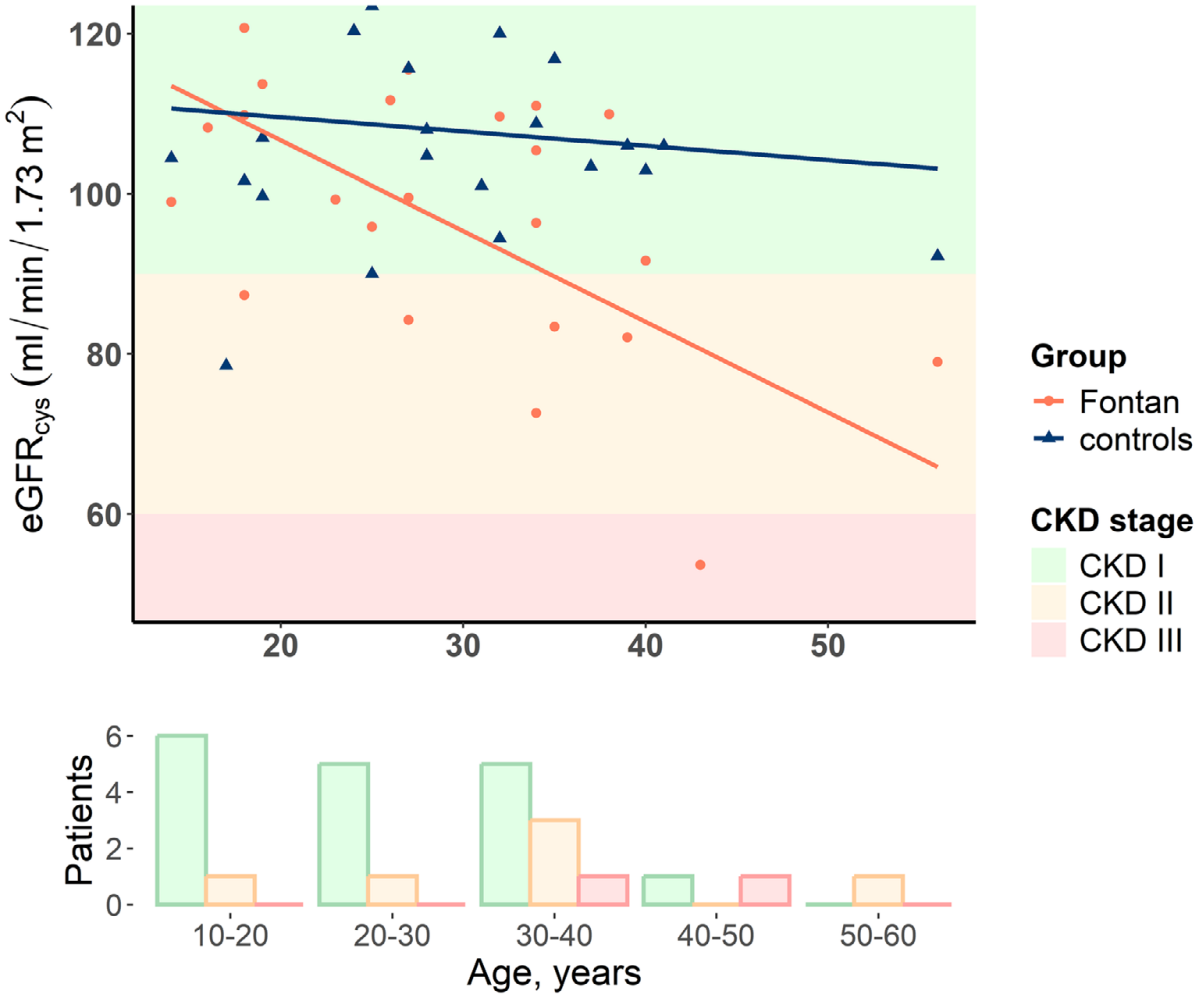
Graphical representation of the association between eGFR_cys_ and age. The orange line represents the correlation line between eGFR_cys_ and age in patients with a Fontan circulation, whereas the blue line represents the correlation in controls. The bottom figure represents the number of patients per CKD group per year group. The colors represent the stages of CKD. Green represents CKD I (eGFR > 90 mL/min/1.73 m^2^), orange CKD II (eGFR 60–90 mL/min/1.73 m^2^), red CKD III (eGFR 30–60 mL/min/1.73 m^2^). CKD = chronic kidney disease, eGFR_cys_ = cystatin C‐based estimated glomerular filtration rate.

We found a negative association between age and eGFR_cys_ in patients (*r* = −0.56; *p* = 0.003) but not in controls (*r* = −0.13; *p* = 0.5) (Figure 2).

We subdivided our cohort by eGFR according to Kidney Disease: Improving Global Outcomes (KDIGO) stages (Table 3). Using eGFR_cr_, 21 patients (84%) could be divided in stage I (eGFR > 90 mL/min/1.73 m^2^) as opposed to 24 controls (96%). Three patients (12%) and 0 controls (0%) could be divided in stage II (eGFR 60–90 mL/min/1.73 m^2^). One patient and one control (4%) could be divided in stage III (eGFR 30–59 mL/min/1.73 m^2^). Using eGFR_cys_, 17 patients (68%) and 24 controls (96%) were subdivided in stage I, 6 patients (24%) and 1 control in stage II, and 2 patients (8%) in stage III.

**TABLE 3:** Prevalence of CKD stages in patients and controls.

	Patients (*n* = 25)	Controls (*n* = 25)	*p*
eGFR_cr_			0.2
CKD I	21 (84%)	24 (96%)	
CKD II	3 (12%)	0 (0%)	
CKD III	1 (4%)	1 (4%)	
eGFR_cys_			0.03
CKD I	17 (68%)	24 (96%)	
CKD II	6 (24%)	1 (4%)	
CKD III	2 (8%)	0 (0%)	

*Note:* CKD I = eGFR > 90 mL/min/1.73 m^2^; CKD II = eGFR 60–90 mL/min/1.73 m^2^; CKD III = eGFR 30–60 mL/min/1.73 m^2^.

Abbreviations: CKD = chronic kidney disease, eGFR_cr_ = creatinine‐based estimated glomerular filtration rate, eGFR_cys_ = cystatin C based estimated glomerular filtration rate.

When assessing the degree of chronic kidney disease (CKD) (i.e., an eGFR < 60 mL/min/1.73 m^2^ or an eGFR > 60 mL/min/1.73 m^2^ in the presence albuminuria), no healthy controls had CKD, seven patients could be classified as having CKD with eGFR_cys_ of whom only one had an eGFR > 90 mL/min/1.73 m^2^, and six with eGFR_cr_ of whom three had an eGFR > 90 mL/min/1.73 m^2^.

### 3.3 24‐h Urine Markers of Kidney Injury

Fontan patients had higher 24‐h urine albumin excretion (14 vs. 2 mmol, *p* < 0.001) than controls. Five patients (22%) had albuminuria (urine albumin > 30 mg/24 h) as opposed to zero controls. There was no difference in 24‐h urine cystatin C (0.034 vs. 0.037 mg/mL, *p* = 0.5) and L‐FABP (2402 vs. 2401 pg/mL, *p* = 0.6) between patients and controls (Table 2).

**FIGURE 3:**
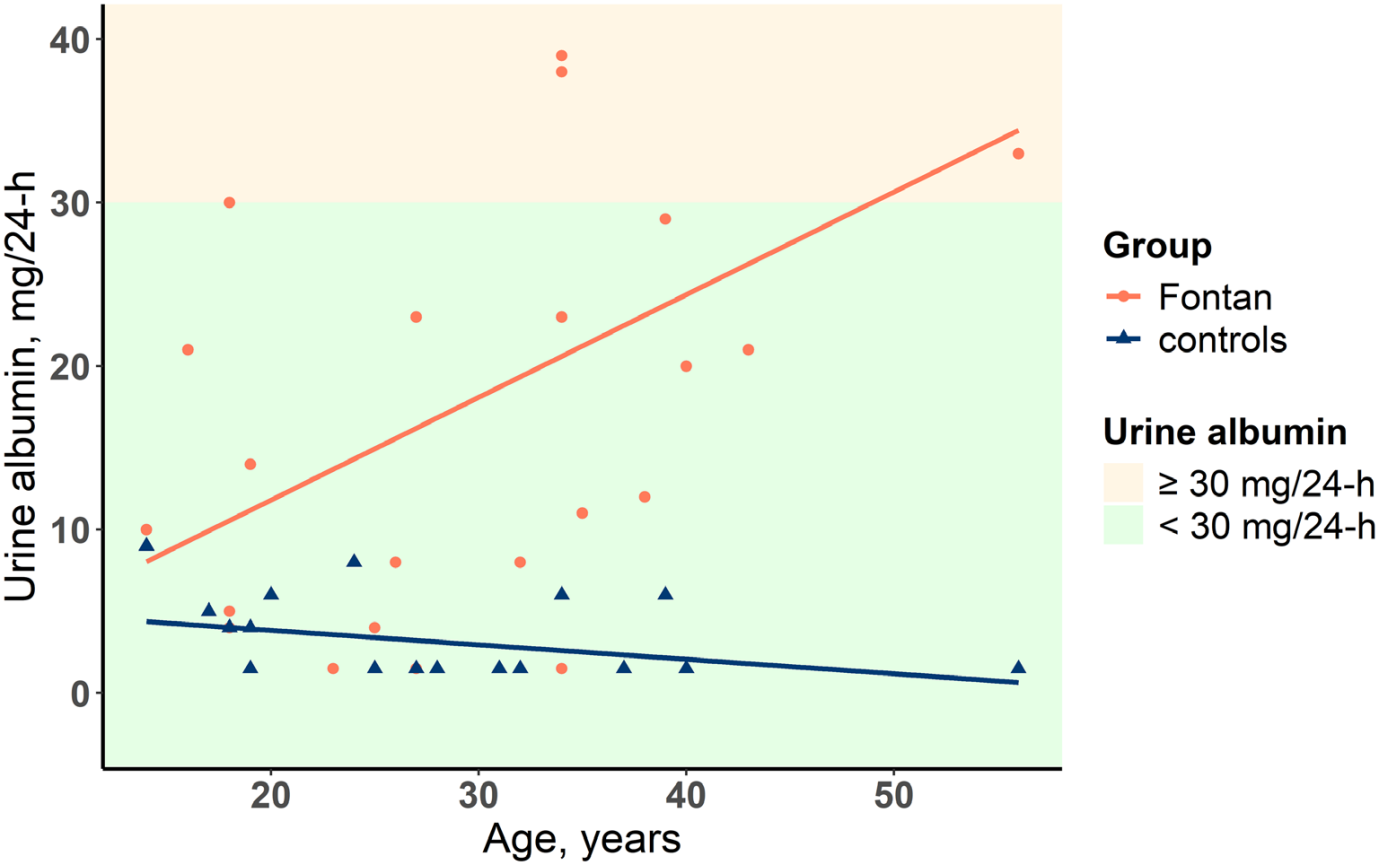
Graphical representation of the association between 24‐h urine albumin and age. The orange line represents the correlation line between eGFR_cys_ and age in patients with a Fontan circulation, whereas the blue line represents the correlation in controls. The colors represent the definition of albuminuria. Green = urine albumin < 30 mg/24‐h = no albuminuria. Red = urine albumin ≥ 30 mg/24‐h = albuminuria.

We found that age was associated with 24‐h urine albumin in patients (*r* = 0.44; *p* = 0.03) but not in controls (*r* = −0.37; *p* = 0.1) (Figure 3). To assess whether the patient older than 50 years disproportionately influenced the correlation between age and 24‐h urine albumin excretion, a sensitivity analysis was performed excluding this patient. Exclusion of this patient did not change the correlation between age and 24‐h urine albumin excretion (Table S1).

### 3.4 Intrarenal Flow Patterns, Venous Impedance Index, and Renal Resistance Index

**FIGURE 4:**
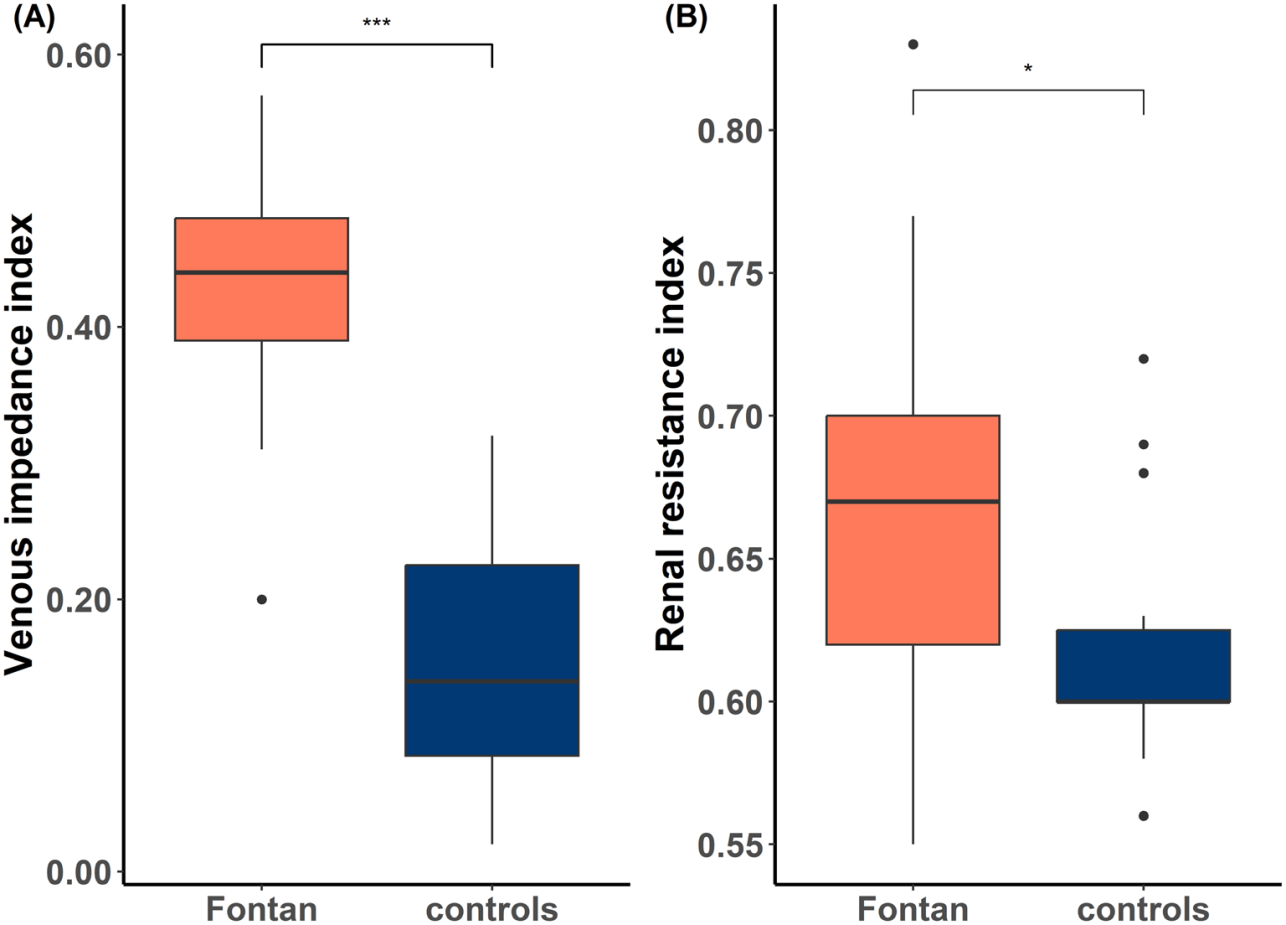
(A) Venous impedance index between patients with a Fontan circulation and healthy controls. (B) Renal resistance index between patients with a Fontan circulation and healthy controls. *p < 0.05. ***p < 0.001.

Twenty four out of 25 patients and 25 controls had a continuous intrarenal venous flow pattern (*p* = 0.9). All patients were in sinus rhythm during Doppler ultrasound imaging. The one patient that did not have a continuous pattern had a bidirectional superior cavopulmonary anastomosis, with the inferior vena cava still connected to the right atrium. We found that patients had a higher VII (0.44 vs. 0.14, *p* < 0.001) compared to controls (Table 4; Figure 4). Additionally, we found that patients with a Fontan circulation have a higher RRI (0.67 vs. 0.60, *p* = 0.02) than controls (Table 4; Figure 4).

**TABLE 4:** Intrarenal flow measurements.

	Patients (*n* = 25)	Controls (*n* = 25)	*p*
Continuous venous flow pattern	24 (96%)	25 (100%)	0.9
RRI	0.67 (0.62–0.70)	0.60 (0.60–0.63)	0.02
VII	0.44 (0.39–0.48)	0.14 (0.08–0.25)	< 0.001

Abbreviations: RRI = renal resistance index; VII = venous impedance index.

We did not find a significant association between age and VII (*r* = 0.30; *p* = 0.1) but we did find a significant association between age and RRI (*r* = 0.41; *p* = 0.04) in Fontan patients.

We further investigated the association between intrarenal flow and kidney function (eGFR_cys_) and injury (24‐h urine albumin, cystatin C, and L‐FABP). We did not find a significant association between VII and eGFR_cys_ (*r* = −0.08; *p* = 0.7), urine albumin (*r* = 0.06; *p* = 0.8), urine cystatin C (*r* = −0.30; *p* = 0.2), or urine L‐FABP (*r* = 0.24; *p* = 0.3).

**FIGURE 5:**
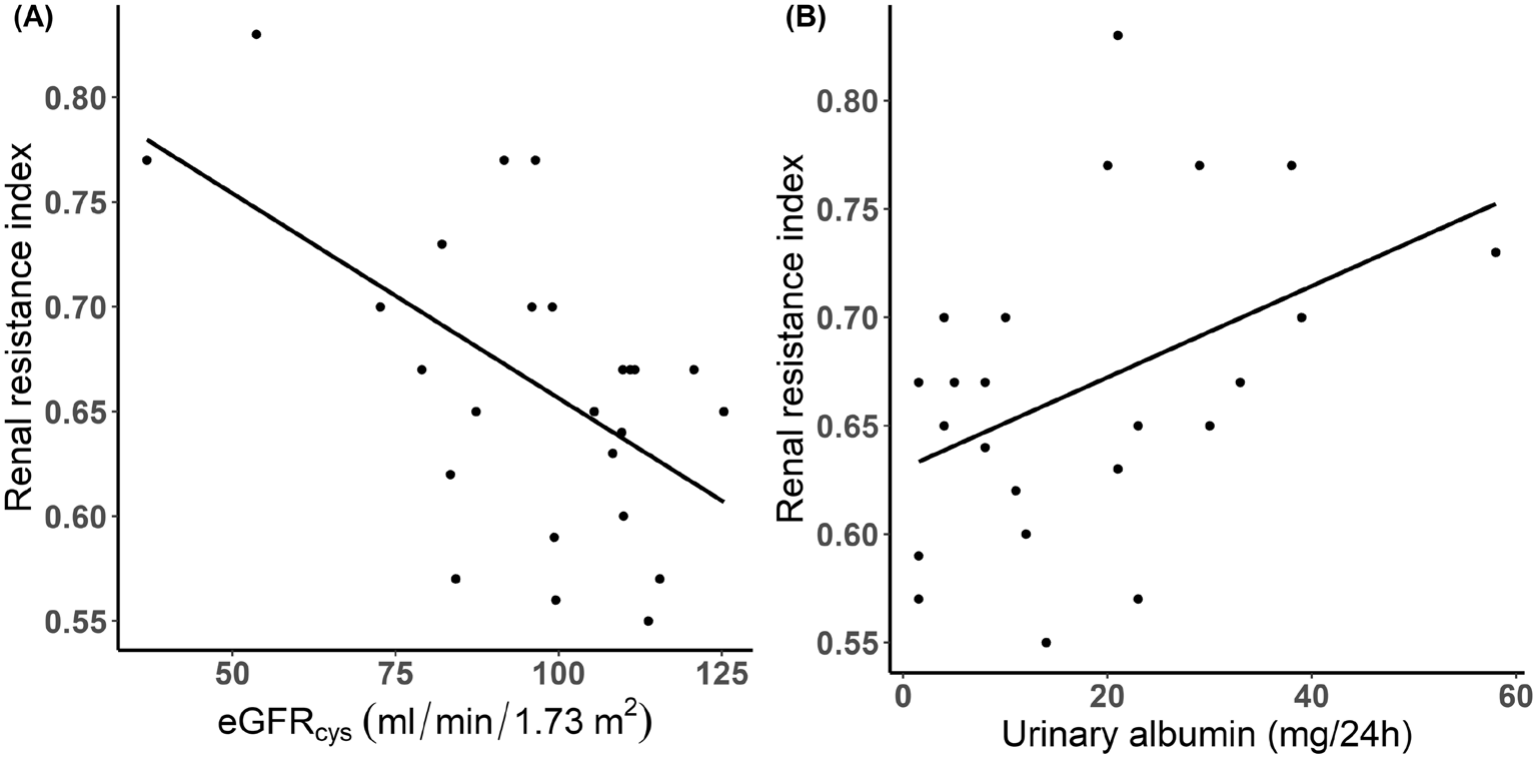
(A) The relationship between RRI and cystatin C‐based eGFR in patients with a Fontan circulation. (B) The relationship between RRI and 24 h urine albumin excretion in patients with a Fontan circulation. RRI = renal resistance index.

We did observe a significant relationship between RRI and eGFR_cys_ (*r* = −0.43; *p* = 0.03) and urine albumin (*r* = 0.43; *p* = 0.04). In order to asses a potential driving effect of the two patients with an eGFR < 60 mL/min/1.73 m^2^ on the correlation between eGFR_cys_ and RRI we performed a sensitivity analysis excluding these patients. Excluding these patients did not change the correlation between eGFR_cys_ and RRI (Table S2). Multivariable linear regression analyses showed that higher age and RRI were associated with lower eGFR_cys_ (St. *β* = −0.47; *p* = 0.018 and St. *β* = −0.48; *p* = 0.015, respectively), independent of sex and VII (Table 5). Additionally, we found that higher RRI was associated with higher 24‐h urine albumin (St. *β* = 0.50; *p* = 0.044), independent of sex and VII (Table 6; Figure 5).

**TABLE 5:** Multivariable linear regression analysis for eGFR_cys_.

	St. β	95% CI	*p*
Age, years^a^	−0.47	−0.85 to −0.09	0.018
Female sex	0.47	−0.27 to 1.21	0.2
RRI	−0.48	−0.85 to −0.10	0.015
VII^a^	0.22	−0.15 to 0.56	0.2

Abbreviations: CI = confidence interval, eGFR_cys_ = cystatin C based estimated glomerular filtration rate, RRI = renal resistance index, VII = venous impedance index.

^a^ Variables were log_2_ transformed.

**TABLE 6:** Multivariable linear regression analysis for 24‐h urine albumin.

	St. β	95% CI	*p*
Age, years^a^	0.09	−0.35 to 0.55	0.7
Female sex	−0.33	−1.31 to 0.64	0.5
RRI	0.50	0.02–0.97	0.044
VII^a^	−0.12	−0.58 to 0.33	0.6

Abbreviations: CI = confidence interval, RRI = renal resistance index, VII = venous impedance index.

^a^ Variables were log_2_ transformed.

We did not find a relationship between RRI and urine cystatin C (*r* = −0.26; *p* = 0.2) or urine L‐FABP (*r* = −0.09; *p* = 0.7). Additionally, we did not find a correlation between RRI and VII (*r* = 0.2; *p* = 0.3). There were no associations between VII or RRI with age or between eGFR_cys_ and kidney injury markers in controls.

Adjustment for the use of angiotensin converting enzyme inhibitors or angiotensin II receptor blockers did not materially alter the results of the association analyses. Additionally, as a large proportion of patients used sildenafil, which lowers pulmonary vascular resistance and consequently CVP, we examined whether sildenafil use was associated with kidney function/damage, RRI, or VII. No significant associations were observed between sildenafil use and any of these variables. The results of these sensitivity analyses are depicted in Tables S3–S16.

## 4 Discussion

The present case–control study was designed to provide a comprehensive phenotyping of Fontan‐associated renal disease. By analogy to Fontan Associated Liver Disease (FALD), we propose the term FARD for Fontan‐associated renal disease in the future. For this purpose, we investigated eGFR, glomerular and tubular injury markers, as well as intrarenal Doppler‐flow patterns in patients with a Fontan circulation, which we compared to healthy age‐ and sex‐matched controls. We found that eGFR_cr_ masks kidney dysfunction in patients with a Fontan circulation as their lower‐than‐expected muscle mass leads to an overestimation of eGFR_cr_ values. This was supported by our findings where patients showed lower eGFR_cys_ levels and reduced creatinine clearance compared to controls. In addition, patients had elevated 24‐h urine albumin levels without a corresponding increase in 24‐h urine cystatin C and L‐FABP, suggesting glomerular injury in the absence of tubular injury. Of interest, despite the obligatory increased CVP associated with the Fontan circulation, venous intrarenal flow patterns were continuous in all but one patient. When assessing continuous rather than dichotomous evaluations of the intrarenal flow patterns, patients had increased VII and RRI compared to controls. Notably, RRI was also independently negatively associated with both eGFR_cys_ and 24‐h urine albumin excretion, whereas VII was not.

### 4.1 Kidney (Dys)function

In general practice, GFR is typically estimated using glomerular filtration markers, with serum creatinine being the most commonly used (Delgado et al. 2022). In the current study, we showed that creatinine‐based eGFR was similar between patients with a Fontan circulation and matched healthy controls. Serum creatinine stems from the spontaneous conversion of creatinine and phosphocreatine in myocytes and is therefore strongly dependent on age, sex, race, and muscle mass (Groothof et al. 2022). The current study showed that Fontan patients had lower 24‐h urine creatinine excretion than controls, reflecting the previously described reduced muscle mass in this population (Avitabile et al. 2014). This lower muscle mass leads to a decrease in serum creatinine levels, which results in an overestimation of eGFR_cr_. Consequently, this overestimation masks the difference in kidney dysfunction between patients and controls as illustrated by the absence of a difference in eGFR_cr_ and CKD stages between patients and controls. In contrast, compared to controls, patients had lower eGFR_cys_ levels, which are estimated using cystatin C—a biomarker less influenced by muscle mass, age, sex, or body composition than creatinine (Spencer et al. 2023; Newman and Cystatin 2002). As a result, patients were more frequently categorized into higher stages of CKD. These findings reaffirm that eGFR_cr_ masks kidney dysfunction in patients with a Fontan circulation. Therefore, this study confirms a previously proposed strategy of using muscle‐mass independent markers for GFR estimation. We further observed a negative association between age and eGFR_cys_ in patients with a Fontan circulation, with the rate of decline being more pronounced compared to controls. These findings align with our previous study demonstrating that eGFR_cr_ declines faster over time in Fontan patients than in the general population, underscoring the progressive nature of kidney dysfunction in this cohort (Hassel et al. 2024).

### 4.2 Kidney Injury

Levels of 24‐h urine albumin were significantly higher in patients with a Fontan circulation than in controls, with over 20% of patients presenting with albuminuria, compared to 0% in the control group. Of note, we found that in contrast to controls, 24‐h urine albumin increased with age in patients, highlighting a progressive course of glomerular injury in this population. Glomerular injury generally stems from several factors such as systemic inflammation, vascular endothelial damage, or neurohormonal activation (Khan et al. 2023). These factors, also commonly observed in patients with a Fontan circulation, can lead to morphologic or structural damage to the glomerulus, allowing albumin to leak into the urine (Khan et al. 2023; Ritmeester et al. 2022). The observed correlations between RRI and age, as well as RRI and 24‐h urine albumin further support the notion of progressive glomerular injury over time. RRI reflects intrarenal arterial compliance, with higher values indicating reduced vascular compliance and increased resistances (Darabont et al. 2023). Neurohormonal activation and systemic inflammation both decrease arterial compliance through vascular stiffening (Ritmeester et al. 2022; Iida et al. 2016; Seo et al. 2020). Therefore, an increase in RRI over time with a negative association between RRI and 24‐h urine albumin suggests that vascular stiffening and reduced compliance contribute to progressive glomerular injury.

In addition to glomerular injury, we also investigated tubular injury markers (urine cystatin C and L‐FABP). Notably, these markers were not elevated in our patients compared to controls, and no association with age was observed. This contrasts with the findings of Opotowsky et al. (2017) who reported significantly higher concentrations of tubular injury markers N‐acetyl glycosaminidase (NAG) and kidney injury molecule‐1 (KIM‐1) in patients with a Fontan circulation than in controls. This discrepancy might be explained by the direct release of NAG and KIM‐1 from damaged proximal tubular cells, whereas increases in urine cystatin C and L‐FABP depend on impaired reabsorption or oxidative stress. Consequently, NAG and KIM‐1 may be elevated at an earlier stage of tubular injury in FARD than cystatin C and L‐FABP.

Within the kidneys, the renal cortex contains the glomeruli, from which the tubules descend into the medulla. In congested kidneys, perfusion is primarily diminished in the medulla rather than in the cortex, which would typically put the tubules at more risk for congestion‐related damage than the glomeruli (Komuro et al. 2018; Voors et al. 2014). The observed presence of glomerular damage without significant tubular injury suggests that systemic factors or factors related to cardiac output and arterial perfusion, rather than solely congestion, play an important role in the pathophysiology of FARD.

### 4.3 Intrarenal Flow Patterns, Venous Impedance Index, and Resistance Index

We are the first to show that patients with a Fontan circulation have a continuous intrarenal venous flow pattern, despite an increased CVP inevitably associated with the Fontan circulation. In biventricular heart failure, intrarenal venous flow patterns typically become discontinuous as venous congestion worsens and CVP rises (Iida et al. 2016; Seo et al. 2020; Tang and Kitai 2016). Similarly, in patients with pulmonary hypertension, marked discontinuous flow patterns have been described. Apparently, in patients with a Fontan circulation and associated elevated CVP, this is not the case. Discontinuous intrarenal venous flow patterns are classified as biphasic or monophasic based on the timing of interruptions during the cardiac cycle: biphasic patterns show flow interruptions during both systole and diastole, while monophasic patterns indicate severe congestion with flow limited to diastole (Maaten et al. 2021). These flow patterns are closely linked to fluctuations in right atrial pressure waves during the cardiac cycle thus mirroring right heart hemodynamics (Iida et al. 2016). The key characteristic of the Fontan circulation, however, is the absence of a sub‐pulmonary ventricle. Moreover, in the current Fontan modifications, the cavopulmonary connection operates independently of (right) atrial dynamics. These unique configurations explain why we did not observe discontinuous intrarenal venous flow in the Fontan patients, except in one in whom the inferior caval vein remained connected to a right atrium. As such, intrarenal venous flow patterns do not hold the same clinical significance in patients with a Fontan circulation as in patients with biventricular circulations.

Patients with a Fontan circulation had elevated VII compared to controls, reflecting reduced intrarenal venous compliance (Gewillig and Brown 2016). We hypothesize that lifelong venous congestion results in compression of vessels within the renal parenchyma which limits their ability to expand, reducing venous compliance (Boorsma et al. 2022). Interestingly, whereas venous congestion is often associated with decreased eGFR and kidney injury, we did not find a relationship between VII and either of these outcomes (Mullens et al. 2008). We cannot exclude that this may be related to the relatively young age of the studied patient cohort. The observed relationship of interlobular RRI with eGFR_cys_ and kidney injury independent of VII suggests that systemic venous congestion may not be the predominant factor in the initiation of FARD. Whether this factor adds to FARD in later stages warrants further investigation.

RRI was significantly higher in patients than in controls, indicating decreased intrarenal arterial compliance. In the context of Fontan physiology, an elevated RRI can be explained by a decrease in arterial compliance resulting from arterial vasoconstriction as a response to activation of neurohormonal activation associated with decreased CO, systemic inflammation and chronic hypoxia, and elevated CVP (Darabont et al. 2023; Saiki et al. 2016; Lambert et al. 2013). Yet, in our study, we did not find a relationship between VII and RRI. It should, however, be realized that this could be attributable to the relatively small sample size of our study. Interestingly, one prior study has investigated the relationship between RRI and long‐term outcomes in patients with a Fontan circulation. In this study, Ohuchi and colleagues found similar results to ours where higher RRI was associated with kidney dysfunction and RR being a strong and independent predictor of all‐cause mortality (Ohuchi et al. 2013). Consequently, RRI could be a robust non‐invasive marker of FARD and Fontan circulatory functioning.

## 5 Strengths and Limitations

To our knowledge this is the first study to phenotype FARD and to compare this phenotyping to healthy age‐ and sex‐matched controls. However, since this is a cross‐sectional study and FARD is a progressive condition, we were unable to investigate serial measurements of eGFR_cys_, urine albumin, tubular injury markers and intrarenal flow patterns, which would have provided valuable insights into its progression over time. In addition, we used VII as a continuous variable rather than categorizing it into renal venous flow patterns, and there is currently little knowledge about the clinical significance of VII as a continuous measurement. We did not use measured GFR for the assessment of kidney function, which may have limited the accuracy of renal function evaluation. However, we increased the accuracy of GFR estimation by using cystatin C, thereby reducing the likelihood of overestimating kidney function. Lastly, the relatively limited sample size of our study limits the ability to draw firm conclusions from the data presented in this study.

## 6 Conclusion

Fontan‐associated kidney disease (FARD) is characterized by progressive deterioration of kidney function and glomerular injury, as evidenced by lower eGFR_cys_ and 24‐h urine albumin in patients compared to controls, whereas late markers of tubular injury were still unaffected. eGFR_cr_ masks kidney dysfunction in patients with a Fontan circulation. Therefore, GFR should be assessed using muscle‐mass independent markers such as cystatin C in this population. Although both VII and RRI were elevated in patients compared to controls, the observed association between eGFR_cys_ and urine albumin with RRI independent of VII suggests systemic factors, rather than systemic venous congestion as the early driver of FARD. Lastly, despite elevated CVP in the Fontan circulation, patients retained continuous intrarenal venous flow patterns, probably due to the absence of right heart mechanics in the cavopulmonary connection. This reduces the clinical interpretation of venous flow patterns in these patients compared to patients with a biventricular circulation.

## Author Contributions

G.H., J.P.M., and R.M.F.B. conceived and designed the research. G.H., I.E.B., and J.M.M. performed the ultrasounds. G.H. analyzed the data. G.H., J.M.M., S.J.L.B., R.M.F.B., and J.P.M. interpreted the results. G.H. and I.E.B. prepared the figures. G.H. drafted the manuscript. G.H., J.M.M., I.E.B., E.T.L., B.Z., S.J.L.B., R.M.F.B., and J.P.M. edited and revised the manuscript. G.H., J.M.M., I.E.B., E.T.L., B.Z., S.J.L.B., R.M.F.B., and J.P.M. approved the final version of the manuscript.

## Funding

The authors have nothing to report.

## Conflicts of Interest

The employer of I.E.B. receives speaker fees by Novartis. J.M.M. declares consulting and/or speaking fees to institution from Bayer, Boehringer Ingelheim, Moderna, Novartis, Novo Nordisk, Roche, and Johnson and Johson. The other authors declare no conflicts of interest.

## Supporting information


**Table S1:** Spearman ρ of the correlation between age and urinary albumin with exclusion of patients with age > 50 years.
**Table S2:** Spearman ρ of the correlation between RRI and eGFRcys with exclusion of patients with an eGFRcys < 60 mL/min/1.73m2 from the analysis.
**Table S3:** Linear regression analysis with eGFRcys as dependent variable and VII and ACEi/ARB as independent variables.
**Table S4:** Linear regression analysis with urine albumin as dependent variable and VII and ACEi/ARB as independent variables.
**Table S5:** Linear regression analysis with urine cystatin C as dependent variable and VII and ACEi/ARB as independent variables.
**Table S6:** Linear regression analysis with urine L‐FABP as dependent variable and VII and ACEi/ARB as independent variables.
**Table S7:** Linear regression analysis with eGFRcys as dependent variable and RRI and ACEi/ARB as independent variables.
**Table S8:** Linear regression analysis with urine albumin as dependent variable and RRI and ACEi/ARB as independent variables.
**Table S9:** Linear regression analysis with urine cystatin C as dependent variable and RRI and ACEi/ARB as independent variables.
**Table S10:** Linear regression analysis with urine L‐FABP as dependent variable and RRI and ACEi/ARB as independent variables.
**Table S11:** Linear regression analysis with eGFRcys as dependent variable and sildenafil as independent variable.
**Table S12:** Linear regression analysis with urine albumin as dependent variable and sildenafil as independent variable.
**Table S13:** Linear regression analysis with urine cystatin C as dependent variable and sildenafil as independent variable.
**Table S14:** Linear regression analysis with urine LFABP as dependent variable and sildenafil as independent variable.
**Table S15:** Linear regression analysis with VII as dependent variable and sildenafil as independent variable.
**Table S16:** Linear regression analysis with RRI as dependent variable and sildenafil as independent variable.

## Data Availability

Data will be made available upon reasonable request.
