# Supplementary material for: Phenotyping of Fontan‐Associated Renal Physiology and Disease
Source: Compr Physiol. 2026 Mar 10;16(2):e70116. doi: 10.1002/cph4.70116 (PMC12975410; doi:10.1002/cph4.70116)
Supplement: Supplementary file 1 — Table S1: Spearman ρ of the correlation between age and urinary albumin with exclusion of patients with age > 50 years. Table S2: Spearman ρ of the correlation between RRI and eGFRcys with exclusion of patients with an eGFRcys < 60 mL/min/1.73m2 from the analysis. Table S3: Linear regression analysis with eGFRcys as dependent variable and VII and ACEi/ARB as independent variables. Table S4: Linear regression analysis with urine albumin as dependent variable and VII and ACEi/ARB as independent variables. Table S5: Linear regression analysis with urine cystatin C as dependent variable and VII and ACEi/ARB as independent variables. Table S6: Linear regression analysis with urine L‐FABP as dependent variable and VII and ACEi/ARB as independent variables. Table S7: Linear regression analysis with eGFRcys as dependent variable and RRI and ACEi/ARB as independent variables. Table S8: Linear regression analysis with urine albumin as dependent variable and RRI and ACEi/ARB as independent variables. Table S9: Linear regression analysis with urine cystatin C as dependent variable and RRI and ACEi/ARB as independent variables. Table S10: Linear regression analysis with urine L‐FABP as dependent variable and RRI and ACEi/ARB as independent variables. Table S11: Linear regression analysis with eGFRcys as dependent variable and sildenafil as independent variable. Table S12: Linear regression analysis with urine albumin as dependent variable and sildenafil as independent variable. Table S13: Linear regression analysis with urine cystatin C as dependent variable and sildenafil as independent variable. Table S14: Linear regression analysis with urine LFABP as dependent variable and sildenafil as independent variable. Table S15: Linear regression analysis with VII as dependent variable and sildenafil as independent variable. Table S16: Linear regression analysis with RRI as dependent variable and sildenafil as independent variable. [file CPH4-16-e70116-s001.docx]

Supplementary material

# Sensitivity analyses with the exclusion of potential drivers of the correlation between age and 24-h urine albumin and between RRI and eGFR_cys_

| **Table S1: Spearman ρ of the correlation between age and urinary albumin with exclusion of patients with age > 50 years** | | | |
| --- | --- | --- | --- |
|  | **ρ** | **95% CI** | **P-value** |
| Age | 0.43 | 0.01 to 0.71 | 0.047 |

| **Table S2: Spearman ρ of the correlation between RRI and eGFR_cys_ with exclusion of patients with an eGFR_cys_ < 60 ml/min/1.73m^2^ from the analysis.** | | | |
| --- | --- | --- | --- |
|  | **ρ** | **95% CI** | **P-value** |
| RRI | -0.42 | -0.71 to -0.01 | 0.04 |

RRI = renal resistance index; eGFR_cys_ = cystatin C-based estimated glomerular filtration rate; 95% CI = 95% confidence interval.

| Sensitivity analyses between VII and RRI with kidney function and renal biomarkers adjusted for the use of angiotensin converting enzyme inhibitors or angiotensin II receptor blockers. **Table S3 linear regression analysis with eGFR_cys_ as dependent variable and VII and ACEi/ARB as independent variables** | | | |
| --- | --- | --- | --- |
|  | **St. β** | **95% CI** | **P-value** |
| Venous impedance index | -0.02 | -0.47 to 0.43 | 0.9 |
| ACEi/ARB use | -0.78 | -2.11 to 0.54 | 0.2 |

| **Table S4 linear regression analysis with urine albumin as dependent variable and VII and ACEi/ARB as independent variables** | | | |
| --- | --- | --- | --- |
|  | **St. β** | **95% CI** | **P-value** |
| Venous impedance index | -0.05 | -0.52 to 0.41 | 0.8 |
| ACEi/ARB use | -0.54 | -1.89 to 0.79 | 0.4 |

| **Table S5 linear regression analysis with urine cystatin C as dependent variable and VII and ACEi/ARB as independent variables** | | | |
| --- | --- | --- | --- |
|  | **St. β** | **95% CI** | **P-value** |
| Venous impedance index | -0.33 | -0.77 to 0.10 | 0.1 |
| ACEi/ARB use | -0.17 | -1.46 to 1.11 | 0.8 |

| **Table S6 linear regression analysis with urine L-FABP as dependent variable and VII and ACEi/ARB as independent variables** | | | |
| --- | --- | --- | --- |
|  | **St. β** | **95% CI** | **P-value** |
| Venous impedance index | 0.20 | -0.24 to 0.63 | 0.4 |
| ACEi/ARB use | -0.43 | -1.73 to 0.86 | 0.5 |

| **Table S7 linear regression analysis with eGFR_cys_ as dependent variable and RRI and ACEi/ARB as independent variables** | | | |
| --- | --- | --- | --- |
|  | **St. β** | **95% CI** | **P-value** |
| Renal resistance index | -0.53 | -0.90 to -0.18 | 0.005 |
| ACEi/ARB use | -0.44 | -1.42 to 0.52 | 0.3 |

| **Table S8 linear regression analysis with urine albumin as dependent variable and RRI and ACEi/ARB as independent variables** | | | |
| --- | --- | --- | --- |
|  | **St. β** | **95% CI** | **P-value** |
| Renal resistance index | 0.46 | 0.06 to 0.86 | 0.03 |
| ACEi/ARB use | -0.78 | -1.81 to 0.24 | 0.12 |

| **Table S9 linear regression analysis with urine cystatin C as dependent variable and RRI and ACEi/ARB as independent variables** | | | |
| --- | --- | --- | --- |
|  | **St. β** | **95% CI** | **P-value** |
| Renal resistance index | -0.25 | -0.68 to 0.17 | 0.2 |
| ACEi/ARB use | -0.20 | -1.34 to 0.94 | 0.7 |

| **Table S10 linear regression analysis with urine L-FABP as dependent variable and RRI and ACEi/ARB as independent variables** | | | |
| --- | --- | --- | --- |
|  | **St. β** | **95% CI** | **P-value** |
| Renal resistance index | -0.06 | -0.49 to 0.37 | 0.8 |
| ACEi/ARB use | -0.61 | -1.77 to 0.54 | 0.3 |

| Sensitivity analyses for the association of sildenafil use with either kidney function or renal biomarkers. **Table S11 Linear regression analysis with eGFR_cys_ as dependent variable and sildenafil as independent variable** | | | |
| --- | --- | --- | --- |
|  | **St. β** | **95% CI** | **P-value** |
| Sildenafil | 0.18 | -0.87 to 1.24 | 0.7 |

| **Table S12 Linear regression analysis with urine albumin as dependent variable and sildenafil as independent variable** | | | |
| --- | --- | --- | --- |
|  | **St. β** | **95% CI** | **P-value** |
| Sildenafil | 0.60 | -0.69 to 1.89 | 0.3 |

| **Table S13 Linear regression analysis with urine cystatin C as dependent variable and sildenafil as independent variable** | | | |
| --- | --- | --- | --- |
|  | **St. β** | **95% CI** | **P-value** |
| Sildenafil | 0.10 | -0.96 to 0.15 | 0.9 |

| **Table S14 Linear regression analysis with urine LFABP as dependent variable and sildenafil as independent variable** | | | |
| --- | --- | --- | --- |
|  | **St. β** | **95% CI** | **P-value** |
| Sildenafil | 0.48 | -0.55 to 1.52 | 0.3 |

| **Table S15 Linear regression analysis with VII as dependent variable and sildenafil as independent variable** | | | |
| --- | --- | --- | --- |
|  | **St. β** | **95% CI** | **P-value** |
| Sildenafil | -0.00 | -1.06 to 1.06 | 0.9 |

| **Table S16 Linear regression analysis with RRI as dependent variable and sildenafil as independent variable** | | | |
| --- | --- | --- | --- |
|  | **St. β** | **95% CI** | **P-value** |
| Sildenafil | -0.35 | -1.39 to 0.70 | 0.5 |

eGFR_cys_ = cystatin C-based estimated glomerular filtration rate; L-FABP = liver-type fatty acid binding protein; VII = venous impedance index; RRI = renal resistance index; 95% CI = 95% confidence interval.
